# Evaluation of Stress Tolerance and Fermentation Performance in Commercial Yeast Strains for Industrial Applications

**DOI:** 10.3390/foods14010142

**Published:** 2025-01-06

**Authors:** Anqi Chen, Qiqi Si, Qingyun Xu, Chenwei Pan, Tianzhi Qu, Jian Chen

**Affiliations:** 1 Science Center for Future Foods, Jiangnan University, Wuxi 214122, China; 6230210079@stu.jiangnan.edu.cn (Q.S.); 6230209026@stu.jiangnan.edu.cn (Q.X.); 6230210021@stu.jiangnan.edu.cn (C.P.); 6220202019@stu.jiangnan.edu.cn (T.Q.); jchen@jiangnan.edu.cn (J.C.); 2 State Key Laboratory of Food Science and Resources, Jiangnan University, Wuxi 214122, China; 3 Jiaxing Institute of Future Food, Jiaxing 314050, China; 4 School of Biotechnology and Key Laboratory of Industrial Biotechnology of Ministry of Education, Jiangnan University, Wuxi 214122, China

**Keywords:** yeast strains, fermentation, stress tolerance, ethanol production, metabolic adaptability, industrial application

## Abstract

This study evaluates the stress tolerance and metabolic adaptability of twelve yeast strains, including eleven commercial strains from Wyeast Laboratories and one prototrophic laboratory strain, under industrially relevant conditions. Yeast strains were assessed for their fermentation performance and stress responses under glucose limitation, osmotic stress, acid stress, elevated ethanol concentrations, and temperature fluctuations. Results revealed significant variability in glucose consumption, ethanol production, and stress tolerance across strains. ACY34 and ACY84 demonstrated the highest fermentation efficiency, while ACY19 exhibited exceptional stress resilience, excelling under multiple stress conditions such as osmotic and ethanol stress. The findings highlight strain-specific performance, with some strains suited for high-yield fermentation and others excelling under challenging environmental conditions. These results provide critical insights for selecting and optimizing yeast strains tailored to specific industrial fermentation processes, contributing to improved productivity and product quality in food and beverage production.

## 1. Introduction

The food and beverage industry, particularly in alcoholic beverage production, relies heavily on the durability and metabolic adaptability of yeast strains, predominantly *Saccharomyces cerevisiae*. This species plays an essential role in converting sugars into ethanol and CO_2_ during fermentation, a process that is influenced by numerous stress factors, including high sugar levels, pH fluctuations, osmotic pressure, ethanol toxicity, and temperature variations [1,2]. These stress conditions, which are common during industrial fermentation, can significantly impact yeast viability, fermentation efficiency, and product quality.

To address these challenges, stress-tolerant yeast strains have been developed or selected to improve industrial performance. For example, osmotolerant strains are widely utilized in high-gravity brewing, where elevated sugar concentrations impose significant osmotic stress. Such strains not only enhance ethanol yield but also maintain consistent fermentation kinetics under these extreme conditions [3,4]. Similarly, strains with high ethanol tolerance are advantageous for producing high-strength alcoholic beverages, as they can sustain metabolic activity and cellular integrity in environments with high alcohol content [5,6]. Additionally, strains that efficiently utilize alternative carbon sources or remain metabolically active under glucose-limiting conditions provide distinct advantages in late-stage fermentation, ensuring complete sugar utilization and consistent product profiles [7].

Despite their industrial importance, comprehensive studies on the stress tolerance and fermentation performance of commercial *S. cerevisiae* strains remain limited. Previous investigations have primarily focused on individual stress factors or isolated strains, leaving a significant knowledge gap in the systematic evaluation of strains under multiple industrial stress conditions. For example, studies have highlighted the variability in stress tolerance among brewing strains, but these studies were limited to specific fermentation applications such as ale or lager production [8,9]. By contrast, this study systematically evaluates a diverse collection of yeast strains, including eleven commercial strains sourced from Wyeast Laboratories and a prototrophic laboratory strain (ACY283, derived from FY4 *GAL*+ *HAP1*+), under multiple stressors. These commercial strains represent various fermentation styles, including ale, mead, and witbier production, providing a comprehensive benchmark for evaluating stress tolerance and fermentation performance.

The strains were chosen to address two key objectives: first, to identify strains with superior stress tolerance and metabolic adaptability for large-scale industrial applications, and second, to gain insights into strain-specific mechanisms underlying stress resilience, such as trehalose accumulation and reactive oxygen species (ROS) management. Notably, the inclusion of ACY283 as a reference strain allows for comparative analysis, ensuring that the observed differences in stress tolerance and fermentation performance can be attributed to inherent strain characteristics rather than experimental variability.

This study is among the first to evaluate such a diverse collection of commercial yeast strains under standardized industrial conditions, providing both practical insights and theoretical contributions to the field of industrial microbiology. By identifying strains with robust fermentation capabilities, this work lays the foundation for further optimization through metabolic engineering or adaptive evolution, ultimately benefiting the food and beverage industry by improving fermentation efficiency, product stability, and production scalability.

## 2. Materials and Methods

### 2.1. Yeast Strains

A selection of yeast strains detailed in Table 1 was evaluated. All strains used in this study were confirmed to belong to the species *Saccharomyces cerevisiae*. Eleven commercial strains from Wyeast Laboratories, widely used in alcoholic beverage production, were chosen for their industrial relevance. The laboratory strain ACY283, a prototrophic *HAP1*+ derivative of FY4, was included as a benchmark due to its well-characterized genetic background and ability to grow without nutrient supplementation [10]. The repaired *HAP1* gene and a *GAL*+ phenotype enabled normal heme regulation and galactose metabolism, making it suitable for controlled comparisons in stress and fermentation studies. All strains were inoculated into YPD medium, isolated as single colonies, and preserved in 15% glycerol at −80 °C for consistency.

### 2.2. Media

Yeast strains were cultivated using minimal or rich media under controlled conditions [11]. Minimal media contained 0.67% (*w*/*v*) yeast nitrogen base with ammonium sulfate (Solarbio, Beijing, China) and 2% (*w*/*v*) carbon sources. Rich media included 2% (*w*/*v*) Bacto peptone and 1% (*w*/*v*) yeast extract (OXOID, Basingstoke, UK) with 2% (*w*/*v*) carbon sources. Solid media, prepared with 2% (*w*/*v*) agar (Solarbio, Beijing, China), were poured into 9 cm Petri dishes (SUPIN, Nantong, China) for culturing. Acidic conditions were achieved by adjusting media pH with HCl.

### 2.3. Microscopic Imaging of Yeast Morphology

Yeast strains were grown in YPD medium (1% yeast extract, 2% peptone, and 2% dextrose) to stationary phase and then subcultured in 5 mL of YPD at 30 °C with shaking at 200 RPM. After reaching stationary phase, cultures were diluted 1:10 with sterile distilled water. A 2 µL aliquot of the diluted culture was examined at 400× magnification using an Olympus BX51 microscope (Olympus Corporation, Tokyo, Japan), and images were processed with ImageJ Version 1.53e for clarity.

### 2.4. Measurement of Fermentation Parameters

Yeast strains were inoculated into fermentation media with an initial OD_600_ of 0.1 and grown in 300 mL flasks containing 100 mL of YPD at 30 °C with agitation at 200 RPM. Samples were collected periodically over 48 h to monitor growth, pH, glucose consumption, and ethanol production. OD_600_ was measured at multiple times points using a UV–Vis spectrophotometer (Spectronic 200, Thermo Fisher Scientific, Waltham, MA, USA) after tenfold dilution with sterile water. Residual glucose and ethanol levels were analyzed using a calibrated biosensor (Siemans, Munich, Germany) after filtering 1 mL of the fermentation broth to remove cells. The pH was tracked using a calibrated pH meter (SevenCompact S210, Mettler Toledo, Columbus, OH, USA).

The average rates of glucose consumption (g/L/h) and ethanol production were calculated using the following formulas [12,13]:

(1) $$\mathrm{average}\;\mathrm{glucose}\;\mathrm{consumption}\;\mathrm{rate}=(\sum_{i=1}^{n}\frac{G_{i-1}-G_{i}}{t_{i}-t_{i-1}})/n$$

(2) $$\mathrm{average}\;\mathrm{ethanol}\;\mathrm{production}\;\mathrm{rate}=(\sum_{i=1}^{n}\frac{E_{i-1}-E_{i}}{t_{i}-t_{i-1}})/n$$

where *G_i_* and *E_i_* represent glucose and ethanol concentrations at *t_i_*, and n is the number of intervals.

### 2.5. Measurement of Cell Density and Calculation of Doubling Time

Cell density was measured as OD_600_ using a Genesys 6 UV–Vis spectrophotometer (JINGHUA, Shanghai, China) or a Synergy H1 Hybrid reader (BioTek, Winooski, VT, USA). For plate-based assays, cultures were inoculated at an initial OD_600_ of 0.05, dispensed into 96-well plates (200 μL per well) and sealed with gas-permeable membranes (Research Products International Corporation, Mt. Prospect, IL, USA) to ensure proper aeration. Plates were incubated at 30 °C with double orbital shaking at 559 rpm. Incubation parameters were specified in figures and captions.

Doubling time (*T_d_*) was calculated using the formula:

*T_d_* = *t* × log2/log (*N_t_*/*N*_0_) (3)

where *N*_0_ and *N_t_* are the initial and final cell densities, respectively, during the log phase, and *t* is the time interval.

Stress conditions included osmotic stress (1 M sorbitol in YPD), ethanol stress (10% ethanol in YPD), glucose limitation (0.5% glucose in YP), cold tolerance (4 °C for three days followed with recovery in YPD), and acid tolerance (YPD adjusting to pH 2.2).

### 2.6. Evaluation of Thermotolerance

Yeast strains were grown overnight in YNB medium with 2% glucose at 30 °C. For heat shock, 0.8 mL of culture was transferred to microcentrifuge tubes and incubated at 45 °C for one hour using a thermomixer (BIOER, Hangzhou, China), while control samples remained at 30 °C. Viability after heat shock was determined by plating serial dilutions on YPD agar and counting colony-forming units after 2–3 days of incubation at 30 °C. Each experiment included at least three biological replicates for accuracy.

### 2.7. Intracellular Trehalose Concentration

To measure intracellular trehalose, yeast cultures at stationary phase with an OD_600_ of 2 were supplemented with trehalose to 1 g/L. After incubating at 30 °C with shaking at 220 rpm for two hours, the cells were washed and lysed according to the Megazyme Trehalose Assay Kit. Trehalose was hydrolyzed into glucose by adding 0.5 µL of trehalase to 20 µL of lysate and incubating at 37 °C for 16 h. Glucose levels were then measured using a Glucose Assay Kit (Nanjing Jiancheng, Nanjing, China) and compared against a standard curve to calculate net trehalose content.

### 2.8. Measurement of Reactive Oxygen Species (ROS) Levels

Yeast cultures were grown in YNB medium with 2% glucose to either logarithmic (OD_600_ ≈ 0.5) or stationary (OD_600_ ≈ 2) phase. Cells were then diluted in phosphate-buffered saline (PBS) and aliquoted into 1.5 mL microcentrifuge tubes. Each tube was treated with 1 µL of 5 mg/mL 2′,7′-dichlorodihydrofluorescein diacetate (DCFH-DA) solution and incubated at 30 °C with shaking for one hour to allow intracellular ROS-mediated oxidation. After incubation, cells were pelleted by centrifugation at 6000 rpm for 3 min, washed three times with PBS to remove excess probe, and resuspended in 600 µL of PBS. For fluorescence measurements, 200 µL of each sample was transferred to a 96-well plate, and ROS levels were quantified using a BioTek microplate reader with excitation at 488 nm and emission at 525 nm [14].

### 2.9. Stress Tolerance Assessment

Stress tolerance in yeast strains was assessed using three growth parameters: doubling time, maximum OD_600_, and time to reach log phase, weighted at 0.4, 0.3, and 0.3, respectively. Mean and standard deviation values were calculated from all strains, and z-scores for each parameter were determined using the formula:

z = (X − μ)/σ(4)

where X is the strain’s value, μ is the mean, and σ is the standard deviation. To normalize scores between 0 and 1, the transformation [15]:

Normalized score = (z − min(z))/((max(z) − min(z)) (5)

was applied, adjusting for metrics where lower values indicated higher tolerance (e.g., doubling time). A composite stress tolerance score was calculated by weighting normalized scores as follows:

Composite score = 0.4 × normalized doubling time + 0.3 × normalized OD_600_ + 0.3 × normalized time to log phase (6)

All experiments included at least three biological replicates to ensure the reliability.

### 2.10. Statistical Analysis

A multivariate analysis of variance (MANOVA) was conducted using Wilks’ lambda to assess the differences among yeast strains under various stress conditions, including acid stress at pH 2.2, 10% ethanol stress, and osmotic stress with 1 M sorbitol. The dependent variables evaluated were doubling time, maximum OD_600_, and time to reach the log phase. Data were collected from each strain under these conditions, with each tested in triplicate. The MANOVA was executed in SPSS version 28, structured with the model formula [16]:

MANOVA (doubling time + maximum OD_600_ + time before log phase ∼ Strain)

Wilks’ lambda was utilized to test the null hypothesis that there are no significant differences between the strains. A Wilks’ lambda value closer to 0 suggests a stronger effect, indicating significant differences. The significance of the effects was determined using the F-value and the *p*-value, with a *p*-value less than 0.05 being considered statistically significant.

## 3. Results

### 3.1. Yeast Morphology

**Figure 1:**
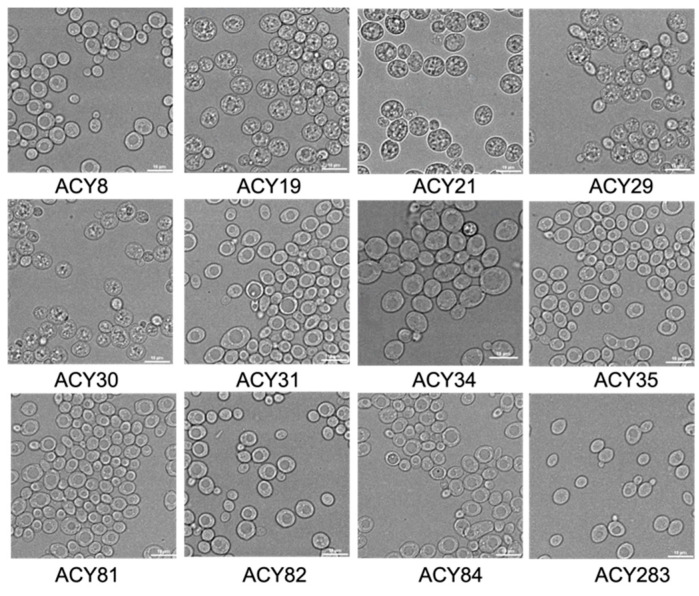
Microscopic analysis of yeast cell morphology. Images were taken at 400× magnification after culturing cells were grown in YPD medium until stationary phase.

Microscopic examination of the commercial ale yeast strains revealed distinct morphological characteristics (Figure 1). ACY8 (London ale) displayed typical ovoid cells with budding patterns, while ACY19 (dry white/sparkling) showed slightly elongated cells. Belgian style strains (ACY21, ACY30, ACY31) exhibited diverse cell sizes and budding formations. ACY29 (sweet mead) had larger, round cells with prominent budding scars. ACY34 (Scottish ale) and ACY35 (Kolsch) presented more compact, round cells. ACY81 (Belgian witbier) and ACY82 (American ale) displayed well-defined, round cells with active budding. ACY84 (Irish ale) demonstrated significant cell clustering.

### 3.2. Fermentation Profile

The fermentation profiles of the tested yeast strains (Table 2 and Supplemental Figure S1) revealed a range of metabolic activities and efficiencies, which can be grouped based on their similar characteristics in terms of glucose consumption, ethanol production, and pH changes.

ACY34 exhibited the highest glucose consumption rate (1.604 g/L/h) and ethanol production rate (0.263 g/L/h), with a minimal pH change (−0.019). Similarly, ACY19 showed a high glucose consumption rate (1.215 g/L/h) and ethanol production rate (0.142 g/L/h), with a slight pH drop (−0.041). Strains like ACY29 and ACY30 also displayed relatively high glucose consumption rates (1.233 g/L/h and 1.193 g/L/h) but lower ethanol production rates (0.108 g/L/h and 0.106 g/L/h) and minimal pH changes (−0.018 and −0.016).

Some strains, such as ACY31 and ACY35, showed moderate glucose consumption rates (1.185 g/L/h and 1.156 g/L/h, respectively) but differed in ethanol production (0.128 g/L/h and 0.173 g/L/h). ACY35 also exhibited a larger pH drop (−0.048), while ACY31 showed a slight pH increase (0.009).

Strains like ACY81 and ACY82 had relatively lower glucose consumption rates (1.178 g/L/h and 1.159 g/L/h) and ethanol production rates (0.057 g/L/h and 0.052 g/L/h), with minimal pH changes (−0.046 for both). ACY283, the laboratory strain, displayed the lowest glucose consumption rate (0.658 g/L/h) and ethanol production rate (0.091 g/L/h), with a very minor pH change (−0.010).

### 3.3. Carbon Source Utilization

The normalized doubling times of the yeast strains across different carbon sources are summarized in Table 3, revealing distinct patterns in their metabolic capabilities. The results highlight the diverse strategies yeast strains employ to utilize various sugars, with notable differences in growth across specific carbon sources. Strains such as ACY8, ACY29, and ACY283 demonstrated consistent and efficient growth across most sugars. For example, ACY29 exhibited robust growth in fructose (0.98 h) and maltose (1 h), while ACY283 performed well across all tested sugars, achieving the highest average normalized doubling time of 0.961. These results suggest that these strains are metabolically versatile and suitable for environments with diverse carbohydrate sources.

Other strains exhibited strong performance in specific carbon sources but lower averages overall due to poor growth on certain sugars. ACY82, for instance, showed excellent growth in glucose (1.00 h) and galactose (1.00 h), outperforming many strains in these conditions. However, it exhibited no growth in maltose and poor performance in trehalose (0.19 h), potentially due to a partial loss of alpha-glucosidase activity. Despite its lower average normalized growth score of 0.645, distinct metabolic traits of ACY82 make it a promising candidate for glucose-rich environments or specific applications, such as stress tolerance studies where it has shown superior performance under cold conditions. Strains such as ACY31, ACY34, and ACY81 displayed limited growth across multiple sugars, with several instances of no measurable growth (e.g., ACY31 in trehalose and sucrose). These strains may benefit from genetic modifications or environmental optimizations to enhance their growth efficiency and metabolic adaptability.

Overall, the variability in doubling times across different carbon sources underscores the importance of assessing strain performance beyond averages. Some strains, like ACY82, excel in specific conditions and warrant further exploration, while others, like ACY283, demonstrate consistent versatility.

### 3.4. Stress Tolerance

#### 3.4.1. Glucose Limitation

**Figure 2:**
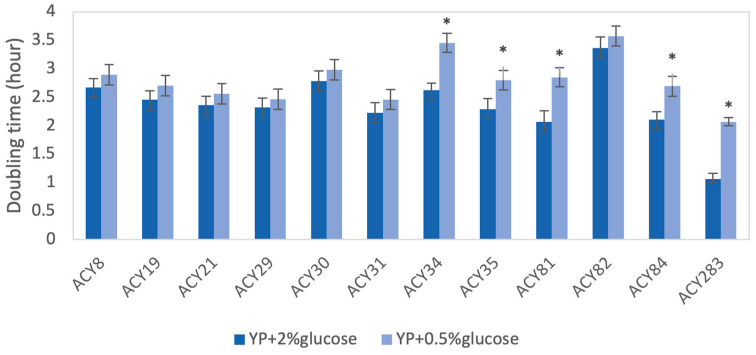
Comparison of doubling times for yeast strains under glucose-rich and glucose-limited conditions. Error bars indicate standard deviations. Statistical significance between the two conditions for each strain is marked with asterisks (* *p* < 0.05).

The impact of glucose concentrations on the growth rates of yeast strains was evaluated under 0.5% (glucose limitation) and 2% glucose (glucose-rich) conditions by comparing their doubling times (Figure 2). Most strains, including ACY8, ACY19, ACY21, ACY29, ACY30, ACY31, and ACY82, exhibited no significant differences in doubling times between the two glucose concentrations. This indicates that these strains have robust metabolic pathways capable of maintaining efficient growth even under glucose-limiting conditions, highlight their adaptability to environments with fluctuating nutrient availability [17]. In contrast, strains ACY34, ACY35, ACY81, ACY84, and the laboratory strain ACY283 showed significantly faster doubling times in the presence of 2% glucose compared to 0.5% glucose (*p* < 0.05 or *p* < 0.01). For instance, ACY81 displayed a pronounced reliance on higher glucose levels for optimal growth, suggesting a metabolic preference for nutrient-rich environments. Similarly, the lab strain ACY283 exhibited significantly slower growth under glucose limitation, reflecting its sensitivity to reduced glucose availability. These observations highlight differences in glucose utilization efficiency among the strains, with implications for industrial processes where glucose availability may vary.

#### 3.4.2. Heat Tolerance

**Figure 3:**
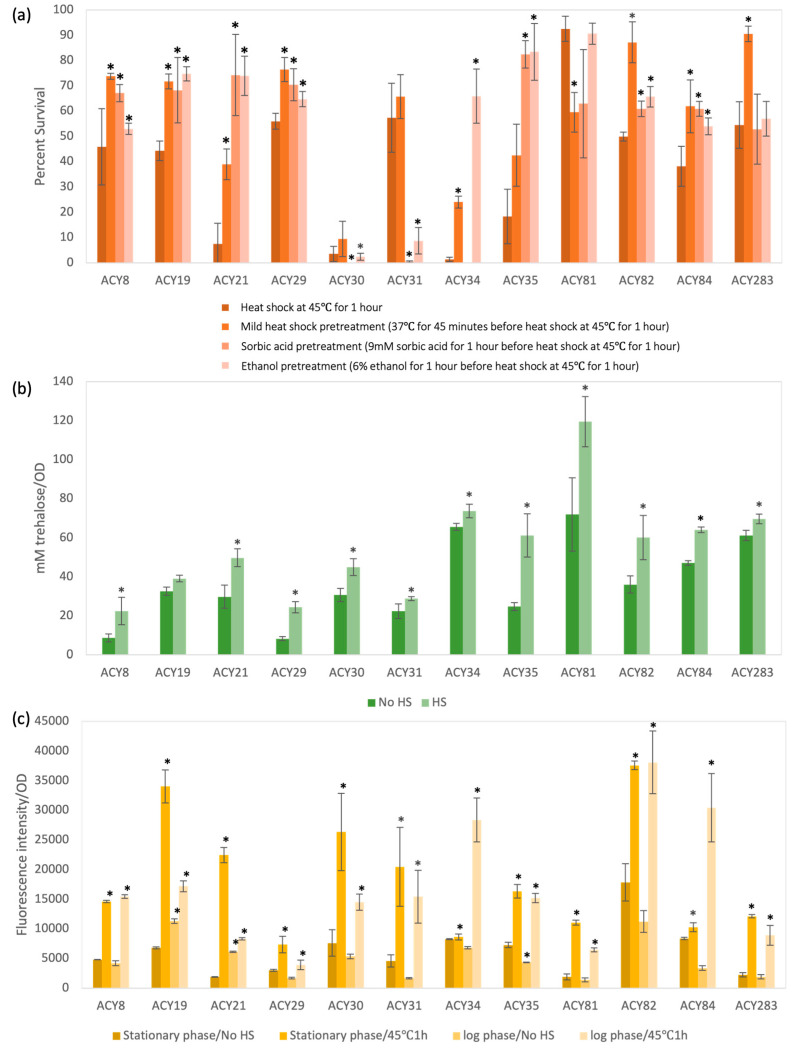
Cellular response to heat shock and protective mechanisms. (**a**) Survival rates of yeast cells after 1 h exposure to 45 °C, with pretreatments including mild heat shock at 37 °C, sorbic acid, and ethanol. (**b**) Intracellular trehalose levels measured before and after heat shock during logarithmic and stationary growth phases. (**c**) Reactive oxygen species (ROS) levels measured pre- and post-heat shock in logarithmic and stationary phases to assess oxidative stress. Error bars represent standard deviations from at least three biological replicates, and significant differences compared to untreated controls are marked with asterisks (* *p* < 0.05).

The heat tolerance of the yeast strains was evaluated under 45 °C heat shock conditions with and without pretreatments, including mild heat, sorbic acid, and ethanol (Figure 3a) [18,19]. Distinct phenotypic groupings were observed. Strains ACY19 and ACY29 exhibited high survival rates across all conditions, including in the absence of pretreatment. For ACY19, mild heat pretreatment further enhanced survival, highlighting its suitability for high-temperature industrial processes such as bioethanol production. In comparison, strains ACY8 and ACY21 showed moderate survival rates that substantially improved with sorbic acid and mild heat pretreatment, suggesting the activation of protective mechanisms under preconditioning [20]. Interestingly, strains ACY34 and ACY35, which showed poor initial survival, exhibited significant improvements when exposed to sorbic acid and ethanol pretreatments, indicating adaptability to preservative-rich environments.

The role of trehalose accumulation was also assessed following heat shock (Figure 3b). Most strains displayed increased intracellular trehalose levels post-treatment. Strains such as ACY19, ACY29, and ACY35 exhibited significant trehalose accumulation coupled with high survival rates, suggesting that trehalose plays a role in their heat stress resilience. Conversely, strains like ACY30 accumulated substantial trehalose without a corresponding improvement in survival, indicating that trehalose alone is insufficient for heat shock protection in some strains [21]. Variations in intracellular ROS levels post-heat shock further revealed strain-specific responses to oxidative stress (Figure 3c). While strains ACY19, ACY29, and ACY35 exhibited elevated ROS levels alongside high survival rates, their ability to withstand oxidative stress implies the presence of effective antioxidant defenses. In contrast, strains such as ACY30 displayed high ROS levels but failed to improve survival, highlighting deficiency in oxidative stress management mechanisms.

#### 3.4.3. Other Stresses

The yeast strains were further evaluated under osmotic stress (1 M sorbitol), cold stress (4 °C for 3 days), acid stress (pH 2.2), and ethanol stress (10% ethanol) using three key parameters: doubling time, maximum OD_600_, and time to enter the log phase. The performance of each strain was normalized and scored to allow for direct comparisons. Results showed significant variability.

Under osmotic stress, ACY19 achieved the highest score (0.727), maintaining a relatively short doubling time (2.12 h) and high cell density (OD_600_ = 1.88) (Table 4). Conversely, strain ACY34 performance the worst, exhibiting delayed growth and lower OD_600_ values. A similar trend was observed under cold stress, where ACY19 again excelled with the highest score (0.968), characterized by rapid growth (doubling time = 1.94 h) and quick log phase entry (9 h). In contrast, ACY30 exhibited a prolonged doubling time (11.39 h) and delayed log phase entry (48 h), suggesting limited cold stress adaptability. Under acid stress, strain ACY19 showed superior performance with a normalized score of 0.934, indicating efficient adaptation to low pH environments. On the other hand, strain ACY34 struggled under acid stress, achieving the lowest score (0.325) due to its slow growth (doubling time = 7.28 h) and delayed adaptation (log phase entry = 17 h).

Under ethanol stress, ACY19 once again demonstrated exceptional resilience, achieving the highest score (0.982) (Table 4). This strain maintained efficient growth (doubling time = 4.34 h) and high cell densities (OD_600_ = 2.09), underscoring its robust metabolic capacity to cope with ethanol toxicity. In contrast, strain ACY82 performed poorly, exhibiting slow growth (doubling time = 19.23 h) and a delayed transition to the log phase (48 h). The overall results reveal that ACY19 consistently outperformed other strains across all stress conditions, highlighting its strong metabolic adaptability and stress tolerance. Strains such as ACY30 and ACY82, which showed poor performance under specific stresses, may require optimization for broader industrial applicability. 

## 4. Discussion

The results reveal notable differences in stress tolerance, metabolic adaptability, and fermentation performance among the evaluated yeast strains, providing critical insights into their potential for industrial applications. The distinct morphological characteristics observed in Figure 1 serve as a foundation for understanding strain-specific physiological traits. For instance, the larger, round cells of ACY29 (sweet mead) and the significant clustering of ACY84 (Irish ale) suggest cellular adaptations that may influence their fermentation efficiency and stress response. These findings align with previous studies that link yeast morphology to metabolic activity and environmental resilience [22,23].

The variability in response to glucose limitation, heat shock, and other stresses highlights the genetic diversity and evolutionary adaptations shaping the metabolic capabilities of these strains. Strains such as ACY8, ACY19, ACY21, ACY29, ACY30, and ACY31 demonstrated robust metabolic pathways, maintaining consistent growth under both glucose-rich and glucose-limited conditions. This adaptability is essential for long-duration or high-gravity fermentations, where nutrient availability fluctuates [24,25]. In contrast, strains like ACY34, ACY35, ACY81, ACY84, and the laboratory strain ACY283 were more dependent on higher glucose concentrations, suggesting an enhanced capacity for glucose uptake or glycolytic activity, making them ideal for high-yield processes under nutrient-rich conditions. This increased capacity may be attributed to more efficient glucose uptake mechanisms, where glucose transporters such as HXT actively facilitate glucose entry, and subsequent phosphorylation by hexokinase enhances the glycolytic flux [26,27]. The glycolytic pathway converts glucose into pyruvate, producing ATP, which supports continued cell growth and fermentation efficiency [28]. Under higher glucose concentrations, these pathways are activated to optimize energy production, thus supporting enhanced metabolic activity and growth.

Interestingly, some strains exhibited an inability to grow on glucose or fructose, such as ACY8, which showed no growth on fructose, and other strains that struggled with glucose utilization. This phenomenon raises important questions regarding the specific metabolic or transport deficiencies in these strains. The inability to utilize these sugars could be due to defects in sugar transporters, such as the absence or dysfunction of *HXT* (for glucose) or *FHT1* (for fructose) transporters, or mutations in enzymes like hexokinase or fructokinase, which are critical for sugar phosphorylation and subsequent metabolism [29,30]. These findings suggest that while certain strains exhibit strong adaptability to diverse sugars, others may have more specialized or limited sugar utilization profiles, making them suitable for specific industrial contexts where glucose or fructose are less abundant or less crucial.

Stress tolerance assessments further underscore the importance of strain-specific traits. ACY19 emerged as the most stress-tolerant strain, excelling under osmotic, acid, cold, and ethanol stresses. Its superior performance underscores the role of enhanced stress response mechanisms, including trehalose accumulation, antioxidant defenses, and heat shock protein networks [31,32]. This resilience positions ACY19 as an excellent candidate for high-gravity brewing and high-alcohol fermentations, where environmental stress often limits productivity. Interestingly, the trehalose accumulation observed in strains like ACY19, ACY29, and ACY35 correlated with higher survival rates following heat shock, suggesting a role for trehalose in stress mitigation. However, the case of ACY30, which exhibited substantial trehalose accumulation without improved survival, highlights the complexity of stress response pathways and the interplay of multiple factors beyond trehalose [33].

The pH changes during fermentation were observed across strains and were closely tied to the production and consumption of metabolites such as organic acids and ammonium ions [34]. Strains such as ACY34 and ACY84 demonstrated minimal pH fluctuations, indicating their ability to maintain stable metabolic activities and buffering capacity during fermentation. This stability is particularly advantageous for large-scale industrial processes where pH changes can disrupt microbial activity and product quality. Conversely, strains exhibiting greater pH variability may require further optimization or external pH stabilization strategies to ensure consistent performance.

The observed stress tolerance and fermentation characteristics provide valuable insights into the industrial applications of these strains. For instance, ACY34 and ACY84 demonstrated high fermentation efficiency and ethanol production, making them suitable for controlled environments that prioritize yield and stability. Their ability to minimize pH fluctuations during fermentation further supports their application in large-scale processes requiring consistent performance. In contrast, the exceptional stress tolerance of ACY19 and metabolic adaptability make it ideal for challenging conditions, such as bioethanol production or processes involving mixed or fluctuating substrates [35].

Future studies should focus on elucidating the molecular mechanisms underlying these traits, with a particular emphasis on the genetic regulation of stress responses, trehalose metabolism, and ROS management. Advancements in synthetic biology, genome editing, and adaptive evolution strategies offer promising avenues for optimizing these strains further. By leveraging these tools, it will be possible to create more resilient and efficient yeast strains tailored to specific industrial needs, ensuring greater reliability and productivity. Collectively, our study highlights the importance of strain-specific traits in determining industrial performance. By systematically evaluating yeast strains under diverse stress conditions, these findings provide a framework for selecting and optimizing strains to meet the demands of large-scale fermentation processes. The insights gained here contribute not only to a deeper understanding of yeast stress responses but also to the development of practical solutions for improving fermentation efficiency and product quality.

## 5. Conclusions

This study provides insights into the stress tolerance and metabolic adaptability of twelve yeast strains, including commercial and lab strains, under various industrial stress conditions that are highly relevant to fermentation process. The evaluation highlighted significant strain-specific variability in glucose consumption, ethanol production, and pH stability, demonstrating that certain strains are better suited for specific industrial applications. Strains ACY34 and ACY84 exhibited the highest fermentation performance. In contrast, ACY19 demonstrated superior stress tolerance, particularly under varying environmental stress factors such as osmotic stress, ethanol toxicity, and temperature fluctuations, underscoring its potential for challenging fermentation environments. These findings emphasize the importance of selecting yeast strains based on their specific metabolic capabilities and resilience to stressors encountered in industrial fermentation. Further research should focus on uncovering the genetic mechanisms behind these traits, enabling the optimization of yeast strains for improved industrial fermentation processes, ensuring better efficiency and consistent product quality.

## Figures and Tables

**Table 1:** Strains used in this study.

Strain *	Description
ACY8	Wyeast Laboratories-London ale
ACY19	Wyeast Laboratories-Dry white/sparking
ACY21	Wyeast Laboratories-Belgian style ale
ACY29	Wyeast Laboratories-Sweet mead
ACY30	Wyeast Laboratories-Belgian high gravity
ACY31	Wyeast Laboratories-Belgian abbey style ale II
ACY34	Wyeast Laboratories-Scottish ale
ACY35	Wyeast Laboratories-Kolsch
ACY81	Wyeast Laboratories-Belgian witbier
ACY82	Wyeast Laboratories-American ale
ACY84	Wyeast Laboratories-Irish ale
ACY283	S288C (prototrophic *HAP1*+ derivative of FY4)

* All strains used in this study were confirmed to belong to the species *Saccharomyces cerevisiae*.

**Table 2:** Fermentation profile.

Strain	Fermentation Profile		
	Average Glucose Consumption Rate (g/L/h)	Average Ethanol Production Rate (g/L/h)	pH Change (pH_initial_ − pH_final_)
ACY8	1.133 ± 0.078	0.120 ± 0.004	−0.054 ± 0.001
ACY19	1.215 ± 0.013	0.142 ± 0.003	−0.041 ± 0.001
ACY21	1.211 ± 0.019	0.114 ± 0.007	−0.032 ± 0.003
ACY29	1.233 ± 0.033	0.108 ± 0.003	−0.018 ± 0.040
ACY30	1.193 ± 0.017	0.106 ± 0.006	−0.016 ± 0.012
ACY31	1.185 ± 0.046	0.128 ± 0.015	0.009 ± 0.018
ACY34	1.604 ± 0.028	0.263 ± 0.006	−0.019 ± 0.006
ACY35	1.156 ± 0.022	0.173 ± 0.008	−0.048 ± 0.004
ACY81	1.178 ± 0.011	0.057 ± 0.018	−0.046 ± 0.001
ACY82	1.159 ± 0.017	0.052 ± 0.008	−0.046 ± 0.000
ACY84	1.115 ± 0.023	0.233 ± 0.001	−0.020 ± 0.001
ACY283	0.658 ± 0.002	0.091 ± 0.006	−0.010 ± 0.003

**Table 3:** Variation in carbon source utilization.

Strain	Doubling Time							
	YP + 2% Glucose	YP + 2% Fructose	YP + 2% Galactose	YP + 2% Maltose	YP + 2% Trehalose	YP + 2% Sucrose	YP + 2% Raffinose	AVERAGE * (Normalized Growth Score)
ACY8	0.70 ± 0.13	0.00 ± 0.14	0.70 ± 0.16	0.99 ± 0.07	1.00 ± 0.11	0.95 ± 0.15	0.84 ± 0.21	0.862
ACY19	0.36 ± 0.17	0.89 ± 0.03	0.35 ± 0.04	0.96 ± 0.05	0.07 ± 0.16	0.96 ± 0.18	0.45 ± 0.20	0.674
ACY21	0.34 ± 0.13	1.00 ± 0.03	0.55 ± 0.05	0.89 ± 0.05	0.06 ± 0.18	0.95 ± 0.21	0.74 ± 0.19	0.753
ACY29	0.71 ± 0.08	0.98 ± 0.03	0.62 ± 0.05	1.00 ± 0.04	0.55 ± 0.16	0.93 ± 0.22	0.68 ± 0.17	0.912
ACY30	0.61 ± 0.06	0.39 ± 0.04	0.79 ± 0.05	0.81 ± 0.05	0.20 ± 0.07	0.38 ± 0.22	0.94 ± 0.19	0.685
ACY31	0.89 ± 0.05	0.38 ± 0.03	0.93 ± 0.05	0.90 ± 0.05	0.00 ± 0.03	0.00 ± 0.22	0.86 ± 0.20	0.660
ACY34	0.00 ± 0.04	0.13 ± 0.04	0.17 ± 0.05	0.85 ± 0.05	0.77 ± 0.05	0.39 ± 0.20	0.85 ± 0.20	0.527
ACY35	0.91 ± 0.04	0.11 ± 0.04	0.72 ± 0.05	0.60 ± 0.04	0.66 ± 0.06	0.35 ± 0.22	0.00 ± 0.17	0.559
ACY81	0.14 ± 0.03	0.00 ± 0.04	0.00 ± 0.05	0.74 ± 0.05	0.42 ± 0.08	0.36 ± 0.23	0.48 ± 0.18	0.357
ACY82	1.00 ± 0.03	0.6 ± 0.04	1.00 ± 0.05	0.00 ± 0.05	0.19 ± 0.10	0.64 ± 0.21	0.44 ± 0.16	0.645
ACY84	0.83 ± 0.03	0.17 ± 0.04	0.13 ± 0.05	0.90 ± 0.06	1.00 ± 0.12	0.71 ± 0.22	1.00 ± 0.15	0.790
ACY283	0.79 ± 0.24	1.00 ± 0.04	0.63 ± 0.31	0.98 ± 0.21	0.58 ± 0.24	1.00 ± 0.14	0.80 ± 0.16	0.961

* The doubling times represent the growth rates of yeast strains under different carbon source conditions, measured in hours. The “AVERAGE *” column reflects normalized growth scores, where values range from 0 (no measurable growth) to 1 (fastest growth observed across all conditions). Normalization allows for direct comparison of relative growth rates among the strains. The values are presented as mean ± standard deviation. ANOVA analysis across conditions showed significant differences in doubling times (F(11, 87) = 20.367, *p* < 0.0001). “0.00” is highlighted in green to indicate no measurable growth, and “1.00” is highlighted in red to denote the fastest growth.

**Table 4:** Variation in high-osmolarity and cold tolerance.

Strain	Doubling Time (Hour)				Maximum OD_600_				Time Before Enters Log Phase (Hour)				Scoring * (Normalized)				Overall Scoring
	Osmotic Stress	Cold Stress	Acid Stress	Ethanol Stress	Osmotic Stress	Cold Stress	Acid Stress	Ethanol Stress	Osmotic Stress	Cold Stress	Acid Stress	Ethanol Stress	Osmotic Stress	Cold Stress	Acid Stress	Ethanol Stress	
ACY8	1.87 ± 0.10	1.71 ± 0.04	4.81 ± 0.08	6.48 ± 0.01	1.59 ± 0.07	2.35 ± 0.01	1.40 ± 0.04	2.02 ± 0.02	7.5 ± 0.13	27.0 ± 0.06	8.0 ± 0.11	35 ± 0.12	0.608	0.824	0.584	0.830	0.645
ACY19	2.12 ± 0.09	1.94 ± 0.04	2.67 ± 0.07	4.34 ± 0.02	1.88 ± 0.06	2.48 ± 0.02	2.20 ± 0.04	2.09 ± 0.03	5.5 ± 0.13	9.0 ± 0.07	5.0 ± 0.11	28 ± 0.12	0.727	0.968	0.934	0.982	0.790
ACY21	2.68 ± 0.11	5.70 ± 0.06	4.10 ± 0.09	4.14 ± 0.03	1.68 ± 0.07	1.61 ± 0.03	1.79 ± 0.05	0.86 ± 0.04	7.0 ± 0.14	37.0 ± 0.08	6.0 ± 0.12	27 ± 0.15	0.451	0.491	0.731	0.752	0.605
ACY29	2.05 ± 0.11	2.28 ± 0.07	3.78 ± 0.09	4.84 ± 0.05	1.84 ± 0.07	1.90 ± 0.04	1.48 ± 0.05	1.58 ± 0.06	10.0 ± 0.14	24.0 ± 0.10	7.5 ± 0.12	32 ± 0.21	0.524	0.763	0.692	0.813	0.711
ACY30	2.14 ± 0.10	11.39 ± 0.10	4.00 ± 0.09	10.87 ± 0.08	1.65 ± 0.07	0.24 ± 0.07	1.90 ± 0.05	0.80 ± 0.08	11.0 ± 0.14	48.0 ± 0.13	7.0 ± 0.13	48 ± 0.32	0.396	0.000	0.748	0.318	0.402
ACY31	2.21 ± 0.11	1.86 ± 0.12	4.20 ± 0.09	9.07 ± 0.10	2.56 ± 0.08	2.48 ± 0.09	1.76 ± 0.05	0.80 ± 0.11	10.0 ± 0.14	23.0 ± 0.16	7.0 ± 0.19	48 ± 0.17	0.705	0.865	0.707	0.350	0.672
ACY34	2.40 ± 0.12	3.19 ± 0.15	7.28 ± 0.10	4.88 ± 0.13	1.78 ± 0.08	2.17 ± 0.11	1.64 ± 0.06	1.32 ± 0.13	11.0 ± 0.15	32.0 ± 0.19	17.0 ± 0.06	35 ± 0.55	0.369	0.703	0.325	0.716	0.474
ACY35	3.02 ± 0.12	2.10 ± 0.17	5.05 ± 0.10	5.51 ± 0.15	1.59 ± 0.09	1.79 ± 0.13	1.75 ± 0.07	1.23 ± 0.15	12.0 ± 0.16	36.0 ± 0.21	7.0 ± 0.07	37 ± 0.72	0.108	0.666	0.632	0.658	0.529
ACY81	2.30 ± 0.14	1.89 ± 0.17	4.80 ± 0.12	26.46 ± 0.16	1.95 ± 0.10	2.09 ± 0.13	2.09 ± 0.08	0.61 ± 0.16	8.5 ± 0.17	8.0 ± 0.21	6.0 ± 0.09	38 ± 0.21	0.562	0.924	0.721	0.143	0.614
ACY82	3.44 ± 0.16	1.31± 0.15	5.93 ± 0.15	19.23 ± 0.16	2.09 ± 0.12	1.56 ± 0.12	1.71 ± 0.10	0.61 ± 0.14	12.0 ± 0.20	33.5 ± 0.19	8.0 ± 0.12	48 ± 0.31	0.155	0.686	0.539	0.130	0.278
ACY84	2.32 ± 0.15	1.88 ± 0.13	3.19 ± 0.14	4.86 ± 0.14	1.78 ± 0.11	2.08 ± 0.10	0.82 ± 0.09	1.30 ± 0.12	11.5 ± 0.19	28.0 ± 0.17	36.5 ± 0.45	38 ± 0.44	0.367	0.773	0.355	0.284	0.472
ACY283	2.38 ± 0.31	2.18 ± 0.02	3.26 ± 0.12	8.31 ± 0.12	1.89 ± 0.02	2.13 ± 0.12	2.59 ± 0.19	1.28 ± 0.05	7.0 ± 0.25	34.0 ± 0.19	5.0 ± 0.48	36 ± 0.35	0.594	0.723	0.948	0.633	0.769

* MANOVA tests revealed statistically significant differences among yeast strains under various stress conditions. For osmotic stress: Wilks’ lambda = 0.0236, F (34, 31.223) = 8.7487, *p* < 0.0001; for cold stress: Wilks’ lambda = 0.0115, F (34, 31.223) = 12.872, *p* < 0.0001; for acid stress: Wilks’ lambda = 0.0026, F (34, 144.255) =261.79, *p* < 0.0001; and for ethanol stress: Wilks’ lambda = 0.00031, F (34, 144.255) = 871.31, *p* < 0.0001. The slowest measurable growth scores are highlighted in green, while the fastest growth scores are marked in red.

## Data Availability

The original contributions presented in the study are included in the article, further inquiries can be directed to the corresponding author.

## Supplementary Materials

The following supporting information can be downloaded at: https://www.mdpi.com/article/10.3390/foods14010142/s1, Figure S1: Fermentation kinetics of yeast strains.
