# Supplementary material for: Evaluation of Stress Tolerance and Fermentation Performance in Commercial Yeast Strains for Industrial Applications
Source: Foods. 2025 Jan 6;14(1):142. doi: 10.3390/foods14010142 (PMC11720210; doi:10.3390/foods14010142)
Supplement: Supplementary file 1 [file foods-14-00142-s001.zip › foods-3362670-supplementary.pdf]

Supplementary Figure

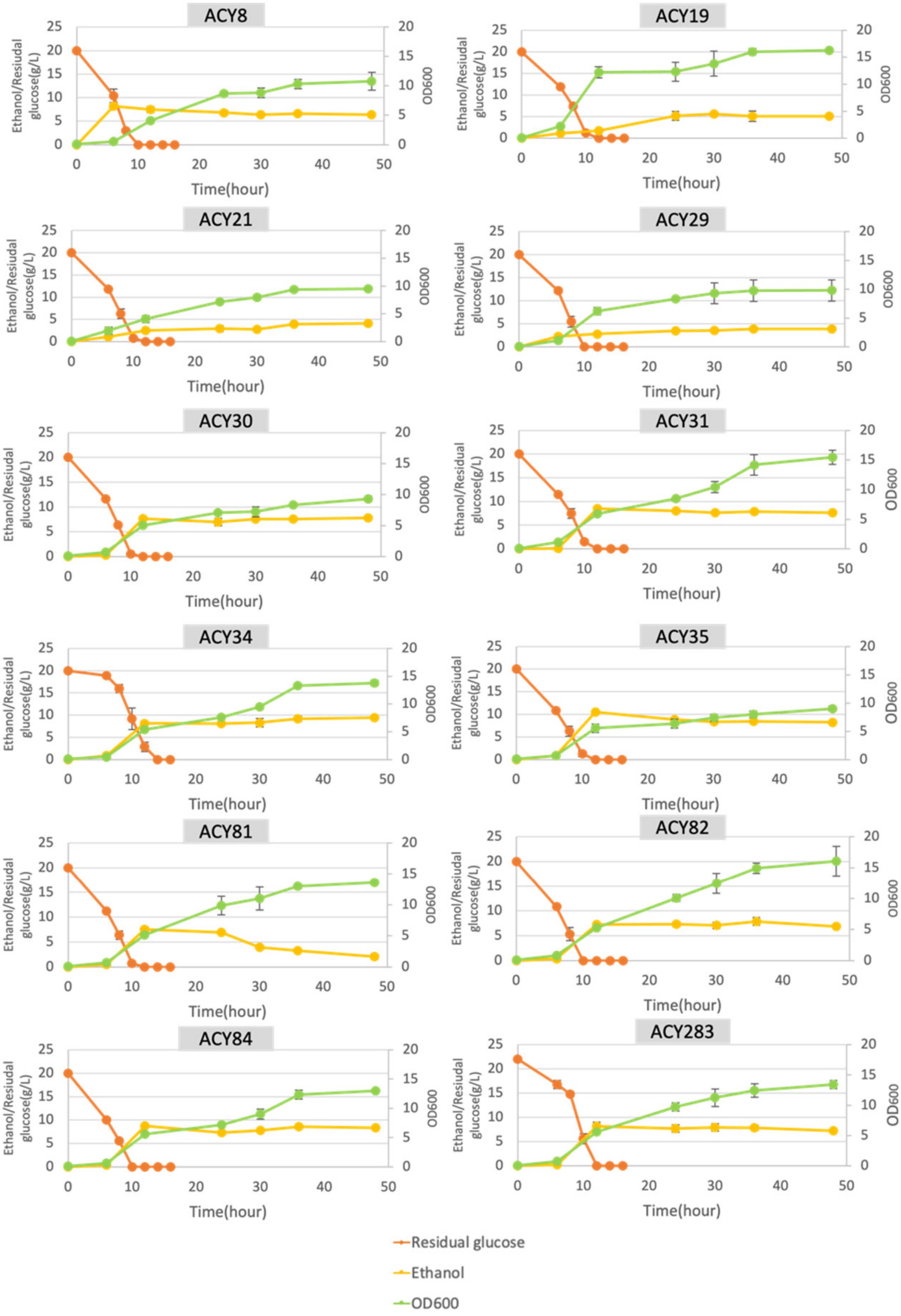

Supplemental Figure S1. Fermentation kinetics of yeast strains. Yeast strains were inoculated at an initial OD_{600} of 0.1 and grown in 100 mL YPD at 30 °C with agitation (200 RPM). Residual glucose was measured at 0, 6, 8, 10, 12, 14, and 16 h, while OD_{600} and ethanol were measured at 0, 6, 8, 10, 12, 14, 16, 18, 24, 30, 42, and 48 h. pH was measured before and after fermentation. Residual glucose and ethanol share the left y-axis (0–25), and OD_{600} is plotted on the right y-axis (0–20).
